# Expression of Conjoined Genes: Another Mechanism for Gene Regulation in Eukaryotes

**DOI:** 10.1371/journal.pone.0013284

**Published:** 2010-10-12

**Authors:** Tulika Prakash, Vineet K. Sharma, Naoki Adati, Ritsuko Ozawa, Naveen Kumar, Yuichiro Nishida, Takayoshi Fujikake, Tadayuki Takeda, Todd D. Taylor

**Affiliations:** MetaSystems Research Team, Computational Systems Biology Research Group, Advanced Computational Sciences Department, RIKEN Advanced Science Institute (ASI), Yokohama, Japan; University of Texas Arlington, United States of America

## Abstract

From the ENCODE project, it is realized that almost every base of the entire human genome is transcribed. One class of transcripts resulting from this arises from the conjoined gene, which is formed by combining the exons of two or more distinct (parent) genes lying on the same strand of a chromosome. Only a very limited number of such genes are known, and the definition and terminologies used for them are highly variable in the public databases. In this work, we have computationally identified and manually curated 751 conjoined genes (CGs) in the human genome that are supported by at least one mRNA or EST sequence available in the NCBI database. 353 representative CGs, of which 291 (82%) could be confirmed, were subjected to experimental validation using RT-PCR and sequencing methods. We speculate that these genes are arising out of novel functional requirements and are not merely artifacts of transcription, since more than 70% of them are conserved in other vertebrate genomes. The unique splicing patterns exhibited by CGs reveal their possible roles in protein evolution or gene regulation. Novel CGs, for which no transcript is available, could be identified in 80% of randomly selected potential CG forming regions, indicating that their formation is a routine process. Formation of CGs is not only limited to human, as we have also identified 270 CGs in mouse and 227 in drosophila using our approach. Additionally, we propose a novel mechanism for the formation of CGs. Finally, we developed a database, ConjoinG, which contains detailed information about all the CGs (800 in total) identified in the human genome. In summary, our findings reveal new insights about the functionality of CGs in terms of another possible mechanism for gene regulation and genomic evolution and the mechanism leading to their formation.

## Introduction

Eukaryotic transcription is a highly complex process typically accomplished by interaction of several proteins and regulatory sequences at different levels to generate a variety of gene products. The ENCODE project recently uncovered complex patterns of dispersed regulation and pervasive transcription for at least 1% of the human genome [1]. Subsequently, the long-standing conventional definition of a gene is fading and it is now realized that the genome is full of overlapping and other complex transcripts [2]. One such intriguing example is the *read-through* transcript or *conjoined* or *co-transcribed* gene (see Table S1 for a list of alternative and proposed names). A “*conjoined gene*” (CG) is defined as a gene, which gives rise to transcripts by combining at least part of one exon from each of two or more distinct known (parent) genes which lie on the same chromosome, are in the same orientation, and often (95%) translate independently into different proteins. In some cases, the transcripts formed by CGs are translated to form chimeric or completely novel proteins. Currently, only 34 CGs are described in the NCBI Entrez Gene database, including well-known examples such as *TRIM6*-*TRIM34* and *NME1*-*NME2* (see http://metasystems.riken.jp/conjoing/faqs#ques2 for a complete list). This “lack of annotation” indicates that this is either a rare phenomenon or that this type of gene has not yet been well characterized in the human genome due to the lack of consensus within the genome annotation community. Also, the use of different gene names to address such transcripts compounds the problem of their identification.

The most widely used resources for accessing human genome annotation information include NCBI Entrez Gene (http://www.ncbi.nlm.nih.gov/sites/entrez?db=gene), the UCSC Genome Browser (http://genome.ucsc.edu/), the Ensembl database (http://uswest.ensembl.org/index.html), and the Vertebrate Genome Annotation (Vega) database (http://vega.sanger.ac.uk/Homo_sapiens/index.html). Even now, seven years after the completion of the human genome, there exist many discrepancies for annotating human genes (including CGs) among these resources, thus somewhat limiting the identification of CGs. For example, CG *FPGT-TNNI3K* is not reported in either NCBI or UCSC although both parent genes, *FPGT* and *TNNI3K*, are present, while in Vega and Ensembl this CG is reported as *TNNI3K* and the parent gene *FPGT* is not present at all. To add to the confusion, the locus representing the *FPGT* gene in NCBI and UCSC is represented as a variant of *TNNI3K* in Vega and Ensembl (see Figure S1). Furthermore, the descriptive contents and the terms used to address CGs across the various resources are not uniform and could be misleading, making it difficult to find and to search for detailed information about them. For example, in plants, read-through transcription commonly refers to genes having multiple polyadenylation sites leading to transcripts that extend for variable distances into the 3′ flanking region of the genes [3]. Confusingly, many known CGs are also termed “read-through transcripts” in these databases. Similarly, “fusion gene” is another misnomer for CGs because the commonly understood definition of a fusion gene is a hybrid gene formed from two previously separate genes that occurs as the result of a translocation, interstitial deletion, or chromosomal inversion. Thus, we propose the term ‘*conjoined gene*’ (CG) for these specialized genes that give rise to transcripts by joining the exons of two or more distinct parent genes during transcription. For clarity, it is important to assign these genes with unique and meaningful names, as is done by the HUGO Gene Nomenclature Committee (http://www.genenames.org/) for all other genes in the human genome.

During the gene annotation of human chromosome 11, eleven CGs were identified by our group [4]. Concurrently, three other research groups independently analyzed the human genome and identified unique CGs using mRNA and EST information available in the public databases [5], [6], [7]. Recently, in two cancer cell lines, MCF7 (breast) and HCT116 (colon), 70 putative CGs were identified using Paired-End diTags [8]. Confusingly, the definition of ‘conjoined genes’ in these analyses is variable, including transcripts formed by combining the exons of two genes in opposite orientations and fused transcripts formed by chromosomal translocations or other rearrangements such as insertions or deletions. Similarly, Li and Zhao et al. [9] recently identified nearly 31,000 “*chimeric RNAs*” in the human genome, however, transcripts formed by CGs were not included in their analysis. According to Denoeud et al. [10], more than 150 CGs were found in the 1% of the human genome analyzed for the ENCODE project; thus, there ought to be many more instances of CGs than have yet been found in the entire genome. Therefore, there is still a need for systematic scanning of the human genome for the identification of more true instances of “conjoined genes”.

In the present study, we report the findings of our “*Conjoin*” algorithm for the identification of CGs in any genome given the availability of its mRNA or EST sequences, along with its reference gene annotation in the NCBI database. Detailed examination of these genes provides useful insights about their roles in the human genome. Furthermore, we have developed a comprehensive database with more uniform and descriptive annotation for the CGs to provide the research community a specialized resource to visualize, examine, and study the characteristics of these genes.

## Results

### Computational identification of CGs

**Figure 1:**
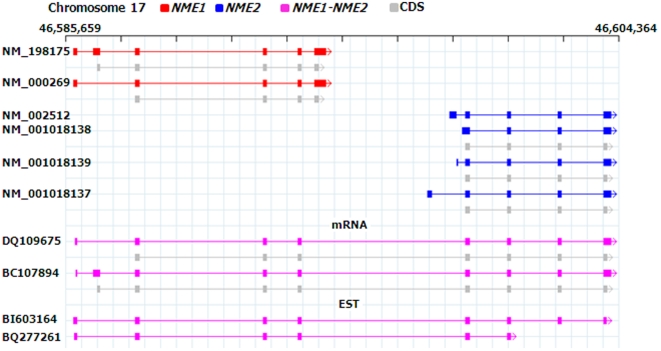
CG formed by parent genes *NME1* and *NME2* on chromosome 17. This CG is found in the NCBI Entrez Gene database (*NME1-NME2*). Two mRNA sequences (DQ109675, and BC107894) and 37 EST sequences (two are shown here, BI603164 and BQ277261) support this CG. The upstream (5′-) and downstream (3′-) parent genes are shown in red and blue colors, respectively. The CG transcripts and CDSs are shown in magenta and grey colors, respectively. Exons and introns are shown as boxes and lines, respectively. Arrows indicate the strand of the transcript.

To estimate the total number of CGs in the human genome, alignments of the known genes, mRNAs, and ESTs to the human genome generated by UCSC (Human assembly (hg18) March, 2006) were used. The algorithm “*Conjoin*” was developed and applied to the entire human genome (see Text S1 for the details). This resulted in 623 and 942 CG candidates from the mRNA and EST data, respectively. In a manual curation step, false positives arising out of misalignments of genes from the same gene family, poor quality sequences, and short EST sequences were removed. We also removed the false positive cases arising from splice variants of single gene loci with multiple gene names (see Figure S2 for an example). As a result we obtained 317 and 434 CGs supported by at least one mRNA or EST sequence, respectively. These 751 CGs connected a total of 1,451 known, separate parent genes, with at least one case found on each chromosome. Interestingly, more than one third of the CGs are formed by parent genes belonging to the functional class of “Cellular Processes and Signaling” as defined in the clusters of orthologous groups for eukaryotes (KOGs) [11] (see Text S2). In addition, 11% (83/751) of the CGs involved parent genes with related functions, such as *TRIM6-TRIM34*, *CCL15-CCL14*, *TNFSF12-TNFSF13*, etc. Figure 1 shows an example of a known CG, *NME1-NME2*, in the NCBI Entrez Gene database on chromosome 17 formed by connecting parent genes *NME1* and *NME2*. Only 12% (88/751) of CGs are supported by more than one transcript, suggesting that these genes are either relatively rare, or that they are not ubiquitously expressed and are limited to only certain tissue types. Interestingly, when multiple transcripts were detected for a CG, alternative splicing was observed in 61% of the cases. Out of the 34 CGs listed in the NCBI Entrez Gene database, 30 were identified by our approach. For the remaining four cases, no aligned mRNA or EST sequences connecting the parent genes were found in the version of data used from the UCSC Genome Browser at the time of this study.

A vast majority (98%) of CGs are formed by only two parent genes, with only a few cases where three (13) or four (4) genes are involved. As expected, the number of CGs per chromosome is significantly correlated with the average gene density and amount of transcriptome data available for each chromosome. However, an inverse correlation was observed for the average distance between genes on each chromosome (see Text S3), implying that genes lying closer on the genome have a higher tendency for forming CGs. Nevertheless, these observations ruled out the possibility of any bias towards certain chromosomes over others.

### Splicing patterns of exons

For the 317 CGs supported by at least one mRNA sequence, we evaluated the exon splicing patterns (see Methods). An interesting pattern was observed in 42% of conjoined mRNAs, where a new intron was created which spans the terminal exon, the 3′-UTR of the upstream (5′-) gene, followed by the intergenic region between the two parent genes, and then the 5′-UTR and initial exon of the downstream (3′-) gene (http://metasystems.riken.jp/conjoing/faqs#ques5). Similar observations were also reported by Akiva et al. [5]. In addition, in 46% of the CGs, novel exons were included from the intergenic or intronic regions, or from the flanking regions of the upstream (5′-) or the downstream (3′-) parent genes. New splice sites have been used in 58% of the CGs; however, in the majority of these cases (85%) splicing occurred at canonical sites (GT-AG). In almost all the CGs (99%), in which splice sites remain conserved from the parent genes, splicing occurred at canonical sites.

### Experimental validation of conjoined genes

**Figure 2:**
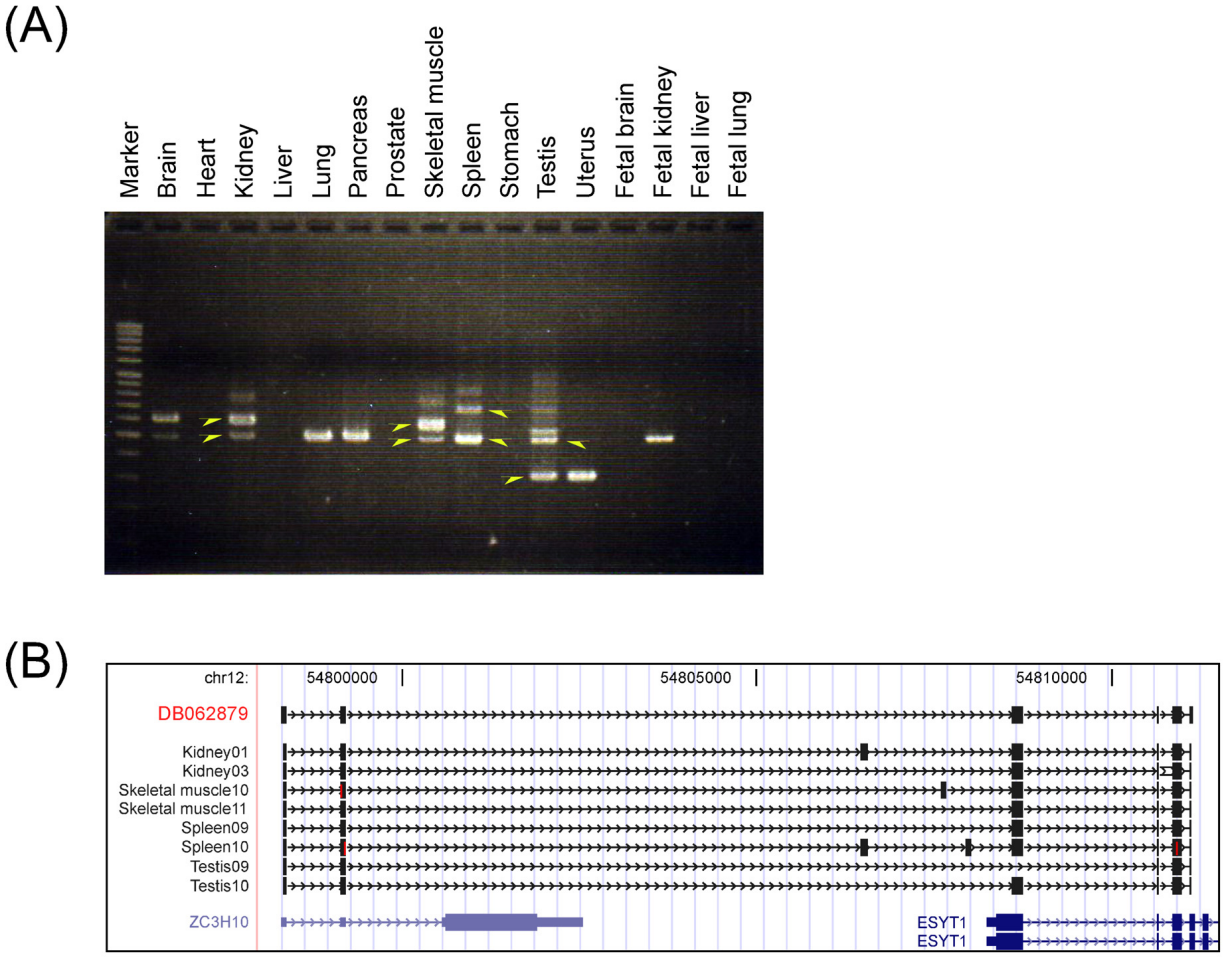
Example of experimentally verified CG, *ZC3H10-ESYT1*, on chromosome 12. (**A**) PCR products amplified from human tissues. This CG (based on EST accession DB062879) was confirmed by RT-PCR in 16 human tissues. It showed various patterns in each tissue. Arrowheads indicate the bands which were cloned and sequenced. Marker: OneSTEP Ladder 100 (0.1–2 kbp) (NIPPON GENE CO., LTD., Toyama, Japan). (**B**) Confirmed CG regions displayed on the UCSC Genome Browser (hg18). The upper tracks (black) represent the seed EST and the identified CG sequences, and the lower tracks (blue) show the RefSeq genes annotated in this region.

We attempted to experimentally validate 353 out of 751 CGs using RT-PCR and sequencing methods in 16 human tissues (see Methods). The CGs in 291 out of 353 (82%) cases were confirmed by sequencing the expected CG transcript in at least one tissue. One representative example is shown in Figure 2. This CG, *ZC3H10-ESYT1*, is supported by one EST sequence (DB062879) in the NCBI GenBank database and was confirmed by sequencing of the RT-PCR product. PCR products of kidney, skeletal muscle, spleen, and testis were selected for sequencing because they contained representative bands of obtained PCR products. Obtained sequences revealed alternative splicing and novel exons, as well as tissue-specific expression. The expected mRNAs could not be sequenced for the remaining 62 CGs. This could be due to non-expression of these genes in the tissues used for validation or because they are expressed at very low and undetectable levels. Interestingly, alternative splicing was observed in 63% (184/291) of the CGs, many of which also harbor novel exons. For the remaining 37% of the CGs, either only a single sequence was obtained by our experiments (for example, CG *PHOSPHO2-KLHL23*), or all of the sequences exhibited the same splicing pattern (for example, CG *TGIF2-C20orf24*), so alternative splicing could not be detected.

These experiments also revealed important information about CG expression patterns. Among the tissues examined, brain and reproductive tissues, including testis, prostate, and uterus, are particularly enriched in CG sequences (http://metasystems.riken.jp/conjoing/faqs#ques11). The reproductive tissues exhibit distinct polyadenylation signal biases [12], which possibly confer them RNA diversity. Mammalian brain has been identified as the tissue that expresses the greatest number of alternative mRNA isoforms [13]. In addition, a large variety of non-coding RNAs have been associated with the central nervous system and impart high complexity to the brain by acting as gene expression regulators [14]. This is likely to be related to the fact that the brain is populated by thousands of highly specialized unique cell types that undergo dynamic changes. Therefore, finding a large number of transcripts encoded by CGs which can increase both protein-coding and non-coding RNA diversity in these tissues is not so surprising, and a similar role of imparting functional diversity and gene expression regulation by CGs cannot be ruled out.

Since 69% (202/291) of CGs were found to be widely expressed (http://metasystems.riken.jp/conjoing/faqs#ques11), formation of CGs is expected to be a more widespread phenomenon occurring in most tissues. However, 31% (89/291) of CGs showed tissue-specific expression, suggesting selective expression for many of them. Certainly, these CGs could be expressed in tissues other than the ones we examined. Using the tissue expression data from the NCBI UniGene database (http://www.ncbi.nlm.nih.gov/unigene), about 76% of the conjoined and participating parent genes were found to be expressed in tissues from various tumors. Although several fusion genes, particularly those produced by chromosomal rearrangements, have been found to be involved in carcinogenesis [15], only a few CGs have been shown to be expressed in cancer tissues, including *RBM6*-*RBM5* [16], *HHLA1*-*OC90* [17], and *LY75*-*CD302* [18]. Therefore, studying the role of these CGs in both normal and cancerous cells in more detail may provide important insights about the diseased conditions.

### Comparison of our findings with those of others

**Figure 3:**
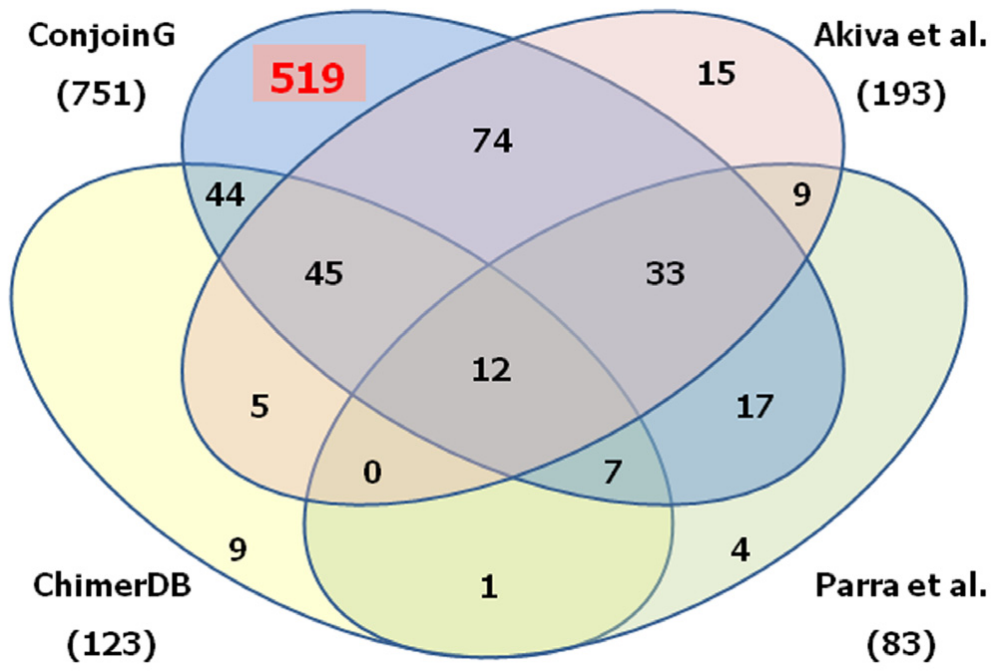
Overlap of the CGs identified by our approach versus those identified by others. Twelve CGs are common among all four datasets. 519 CGs were uniquely identified by our approach. (Akiva et al [5], Parra et al [6], and Kim et al [7] (ChimerDB)).

In early 2006, Akiva et al. [5] and Parra et al. [6] independently analyzed the human genome and identified 212 and 127 CGs, respectively, using mRNA and EST information available in the public databases. At the same time, another analysis done by Kim et al. resulted in the identification of 258 unique CGs in the human genome [7]. On close examination, we found that some of these genes have now become obsolete due to the lack of appropriate alignments of the CG mRNAs with the parent genes, revision of the coordinates of the participating parent genes in the current version of the human genome databases, or different chromosomal location or opposite orientation of the parent genes. As a result, only 193, 123, and 83 CGs remain valid in the Akiva, Kim, and Parra datasets, respectively. To estimate the total number of unique CGs, we compared the 751 CGs identified in this study with these three datasets (Figure 3). 232 out of 751 CGs were included in at least one other dataset, with 106 being confirmed in at least one human tissue by our experiments. The remaining 519 (69%) CGs were uniquely identified by our method only, 185 of which were experimentally validated. Forty-three CGs were uniquely identified by at least one of the Akiva, Kim, or Parra analyses, but not by us.

We also compared the 13 chimeric transcripts obtained by RT-PCR by Denoeud et al. [10] in the ENCODE region to our dataset. Only two were found in common, and five were doubtful by the criteria mentioned above. Six CGs in the ENCODE study were uniquely identified by them. The sequences used to confirm these CGs were not found in the version of the human genome data from UCSC that was used for our analysis. Thus, adding the unique CGs identified by Akiva, Kim, Parra and the ENCODE analysis to ours results in 800 unique CGs identified in the entire human genome to date. This clearly indicates that there may still be many more instances of CGs in the genome and that the existing genomic information is not yet sufficient to identify all of them.

### Functional roles of conjoined genes

#### Protein evolution by chimeric proteins

The significance of gene fusion in eukaryotic evolution has been previously demonstrated [19]. The specialized splicing patterns observed in CGs can result in novel proteins, which may play a role in the additional complexity of the human genome as demonstrated in case of chimeric protein *Kua-UBE2V1* [20]. For open reading frame (ORF) prediction, only those CGs were selected for which at least one mRNA sequence was found and a reasonable ORF could be obtained (297 CGs, 409 mRNAs, see Methods). Transcripts arising from 16% of the selected CGs used conserved reading frames of translation from all the parent genes, thereby forming chimeric proteins by joining the domains of their respective parent genes (Table 1). It is well known in prokaryotes that the vast majority of gene pairs whose orthologs are fused are either part of the same complex, or function in the same pathway [21]. Thus CG formation can result in co-regulation of gene expression of functionally related proteins such as for *TNFSF12-TNFSF13* [22] and *TRIM6-TRIM34* [23].

**Table 1:** Reading frames used for the formation of the CG ORFs.

Reading frames used by CG	Protein Product	% CG (# of CGs/Total Selected)		Examples
		Predicted NMD candidates	Predicted Non-NMD candidates	
Same as all parent genes (conserved)	Chimeric	16% (48/297)		*NME1-NME2*
		5	43	
Same as one of the parent genes (partially conserved)	Similar to one of the parent genes	79% (234/297)		*ARID4B-RBM34, OMA1-DAB1*
		47	187	
Different than all parent genes (different)	Novel or non-coding	5% (15/297)		*DCDC1-DCDC5*
		0	15	

#### Gene regulation by CG expression

Alternatively, CGs can perform other regulatory roles, such as altering the expression of parent genes. They can achieve this by using altogether different frames of translation in the conjoined transcript with respect to all the parent genes, thus preventing their expression by forming a novel protein or non- protein-coding transcript (5% of CG mRNAs). Alternatively, the CG transcript may be translated using the conserved reading frame from only one parent gene. The sequence corresponding to the other parent gene is then either translated in a different reading frame or remains as an untranslated region (79% of CG mRNAs) (Table 1). In either of these scenarios, expression of one of the parent genes is affected. In some mouse tissues, CG *Ankhd1-Eif4ebp3* shows a similar expression pattern as that of *Ankhd1*, whereas the expression of *Eif4ebp3* is significantly lower in these tissues [24]. A similar role of gene regulation is expected for some other CGs such as *INS-IGF2*, *ZFP91-CNTF*, *MUTED-TXNDC5*, etc., in which the CG ORF is similar to only one of the parent gene's ORF. Interestingly, in a slightly larger number of CGs (58%), the predicted ORF was more like that of the downstream (3′-) parent gene as compared to the upstream (5′-) parent gene (42%).

Protein expression, however, is highly controlled by the Nonsense Mediated Decay (NMD) mechanism [25]. Thus, it is not surprising that 18% of CGs are expected to undergo NMD due to the appearance of a premature stop codon as a result of frame-shift caused by altered exon-intron splicing with respect to the parent genes, or by the inclusion of novel exons. Even in these cases, expression of the parent genes may be temporally regulated by the formation of a conjoined transcript.

#### Occurrence of conjoined genes in other genomes

The functional role of CGs can be further strengthened if they are shown to survive through selective evolutionary pressure. Hence, we examined the conservation of CG ‘junction exons’ across 23 other vertebrate genomes using BLAT (e≤10^−6^). The ‘junction exons’ can be defined as the exons which contain DNA sequence from both participating parent genes, that is to say, the last exon of the upstream (5′-) gene and the first exon of the downstream (3′-) gene, in the transcripts formed by the CGs. In cases where the last and first exons of the two parent genes, respectively, form separate exons in the CG transcripts, with or without any novel exons between them, the entire region was used.

More than 70% of the human CG junction exons were found to be conserved across eight other vertebrate genomes (http://metasystems.riken.jp/conjoing/faqs#ques4). No significant conservation was observed in the lower-order vertebrates including zebrafish, *X. tropicalis*, medaka, stickleback, lamprey, tetraodon, and fugu although a large number of mRNA and EST sequences were available for all these organisms. Among the higher-order vertebrates, maximum conservation of CG junction exons was observed in the chimpanzee genome. We observed a large decrease in the number of conserved human CG junction exons from chimpanzee to macaque and orangutan. This could be due to the poor quality of the transcriptome annotation for these genomes. With the advancement in high-throughput transcriptome sequencing technologies, such as RNAseq, more RNA sequence data is expected to be available in the near future, leading to the detection of additional conserved human CGs in these and other genomes. Nevertheless, all other genomes showed much less conservation of human CG junction exons. Therefore, it is evident from our analysis that CG conservation does not depend on the amount of sequence data available for any given genome; instead, it is correlated with the order of complexity of the vertebrate genomes.

Conservation of CGs across several different vertebrate genomes implies that their formation is not only limited to human. To further explore this possibility, we applied the ‘Conjoin’ algorithm on three eukaryotic genomes namely, mouse, fruit fly, and dog. Interestingly, 270 and 227 CGs were identified in mouse and fruit fly respectively, whereas no CGs could be detected in the dog genome using the currently available mRNA and EST data from UCSC. The junction exons from the mouse and fruit fly CGs were extracted as described above for human CGs, and were searched in the human mRNA and EST datasets using BLAT (e≤10^−6^). Only 0.03% of the mouse CG junction exons were found conserved in human, whereas no fruit fly CG junction exons were detected. Identification of CGs in the mouse and fruit fly genomes further emphasizes that CGs are not mere artifacts of the transcription process, but that they likely have well-defined roles in either gene regulation or protein complexity or both.

### Identification of CGs from the regions where no prior CG transcript evidence was available

We found that the median distance between parent genes forming CGs is 10 kb, except for a few outliers such as *DOCK5-PPP2R2A*, *LASP1-PPP1R1B*, *FIP1L1-PDGFRA*, and *MATR3-PURA*, which are formed by parent genes that lie as far apart as 700–800 kb, bypassing exons of several internally located genes. Since the median distance between genes in the entire human genome is roughly 64 kb, this clearly indicates that CGs are preferentially formed by genes lying much closer to each other. In total, there are about 3,000 gene pairs, arising from 5,050 unique genes (20% of all the known human genes), which lie less than 10 kb apart on the same strand that could potentially encode CGs. Therefore, the possibility of finding more CGs in the human genome cannot be ruled out.

To test this possibility, we randomly selected ten pairs of genes (test cases) from the human genome which satisfied the following ‘minimum’ criterion for formation of CGs; they are (i) on the same chromosome, (ii) on the same strand, and (iii) less than 10 kb apart. Since the most common pattern of CG splicing is to exclude the terminal exon of the upstream (5′-) gene and the initial exon of the downstream gene (3′-), we designed PCR primer sequences for these regions from the second-to-last exon of the upstream (5′-) gene and the second exon of the downstream (3′-) gene. We then performed RT-PCR, followed by sequencing, of the positive PCR fragments. Surprisingly, for 80% (eight out of ten) of the test cases, we successfully verified the expected CG mRNA joining the two parent genes (Table 2). Like most other CGs, alternative splicing with the inclusion of novel exons was also observed in these test cases. This clearly indicates that more than 20% of the human protein-coding genes are capable of forming CGs, and the formation of CGs is an innate process in the genome which does not necessarily take place under any special conditions such as the diseased state. However, the existing transcript information for the human genome is still not comprehensive enough to detect all possible gene variants and there lies the great possibility of finding many more CGs and other novel genes [26] using other more sophisticated procedures.

**Table 2:** Ten test cases in human for which no prior evidence existed.

Upstream (5′-) Gene Symbol	Downstream (3′-) Gene Symbol	Strand	Chromosome	CG Confirmed?	Alternative Splicing Detected?
*ACTG2*	*DGUOK*	+	2	Yes	Yes
*ABHD14A*	*ACY1*	+	3	Yes	Yes
*SELK*	*ACTR8*	−	3	Yes	Yes
*ABCG4*	*NLRX1*	+	11	Yes	Yes
*ACAD10*	*ALDH2*	+	12	Yes	Yes
*ACCN2*	*SMARCD1*	+	12	Yes	No
*ING4*	*ACRBP*	−	12	Yes	Yes
*DPM1*	*ADNP*	−	20	Yes	Yes
*SIGLEC1*	*ADAM33*	−	20	No	NA
*PPAT*	*AASDH*	−	4	No	NA

### Development of ConjoinG: Database of Human Conjoined Genes

Because of the lack of uniformity in the annotation of CGs across the various human genome resources (UCSC, GenBank, Ensembl, Vega), we realized that there was a need for a dedicated and comprehensive repository for such genes. This repository could also act as an important resource to link the information about these genes from the other databases. Therefore, we developed a comprehensive database, called ConjoinG, which harbors detailed information about each of the 751 unique human CGs identified by our method and 49 CGs identified by other approaches. The ConjoinG database has several useful functionalities such as visualization of the CG mRNAs and ESTs with respect to the parent genes in their genomic context, similarity of the CG coding DNA sequences (CDSs) with those of the parent genes, conserved regions of the CG ‘junction exons’ in other vertebrate genomes, etc. In addition, the database harbors details about the experiments used to confirm the CGs, including primers used for RT-PCR, sequences of the PCR products mapped back on the genome, tissues in which the CGs were found expressed, and so on. This database also includes several simple and advanced query options and can be accessed freely at http://metasystems.riken.jp/conjoing/.

## Discussion

The mammalian transcriptome is much more complex than previously thought. Several recent studies suggest that most of the mammalian genome is transcribed, yet thousands of transcripts do not encode for proteins [27]. These non-protein-coding genes, along with some CGs, play a variety of regulatory functional roles. In this analysis we report the identification of 751 CGs, and for the first time experimental confirmation of the existence of 82% (291 out of 353 representatives) of CGs in 16 human tissues. Some of the CGs also overlapped with those identified in other studies (Figure 3), but a large majority of them were uniquely identified by our method only.

Recently, induced chromosomal proximity has been shown to give rise to gene fusions in some cancer cells [28]. Chromosomal folding is known to bring loci which are far apart in the linear sequence in close proximity in the three dimensional space of the transcription factories [29]. Thus, the likely role of chromosomal folding, giving rise to distinct loops, for the formation of some CGs cannot be ruled out. Based on physical properties, the estimated minimum length of a typical chromatin loop is more than 10 kb; however, shorter loops are also possible [29]. Interestingly, most parent genes resulting in the formation of CGs are found to be about 10 kb apart. Thus, chromatin loop formation could bring the parent genes in close proximity in the transcription factories where they may form the CG transcript. In a few recent studies, two different models have been proposed for the formation of chimeric RNAs (not including CGs) in the transcription factories. First, the SHS (short homologous sequence) mediated “transcriptional slippage” model has been described for the formation of interchromosomal chimeric RNAs [9]. Only a very small fraction of CGs (10%) were found to harbor such SHSs (≥4 bps) at their junction. In the second model, “trans-splicing” mediated formation of chimeric RNAs was proposed [30], [31]. However, both of these models suffer from their own limitations. In the “transcriptional slippage” model, the presence of an SHS element at the junction seems essential; whereas in the “trans-splicing” model, the appearance of novel exons found in many of the CGs cannot be explained. Thus, a single model is not sufficient to explain the formation of CGs.

From our observations, we propose a third mechanism of “transcriptional run over and intergenic splicing” to be operating, especially in the formation of CGs which harbor novel exons from the intergenic regions. In our proposed mechanism, the transcriptional machinery first transcribes the upstream (5′) gene but somehow escapes its termination signal, it then continues transcribing the downstream (3′-) gene as part of the same conjoined transcript. This escape from termination of the upstream (5′-) gene seems unlikely to be inadvertent; rather, it appears to be well-regulated by some yet unknown trans-factors, as cis-regulatory elements, except for simple repeats, showed no unusual trends in the flanking regions of CGs (see Text S4). In prokaryotic genomes, several transcription anti-termination factors are known which help in the escape of the termination signals and consequently lead to transcriptional read-through into the next gene [32]. The identification of CGs suggests the presence of similar factors in eukaryotic genomes as well, which are less well explored to date. In addition to anti-termination factors, the presence of multiple polyadenylation sites might also aid in the formation of CGs. Recently, the use of alternative polyadenylation sites has been realized as another mechanism for generating a variety of mRNA transcripts [13]. The choice of polyadenylation site is found to be affected by several factors, such as tissue specificity or differing cell types [13], [33]. Also, it has been demonstrated that almost half of the genes in the human genome are alternatively polyadenylated [34]. Therefore, the formation of CG transcripts, with the help of yet unidentified anti-termination factors, in addition to alternative polyadenylation, appears likely and requires further investigation.

Since it is evident that CGs are not merely artifacts of transcription, then they must be the result of some specific genomic requirements and have well-defined functional roles. This idea is further strengthened by the fact that some CGs have endured purifying evolutionary selective pressure and are conserved in other vertebrate genomes such as chimpanzee, macaque, mouse, and dog. In addition, formation of CGs is not only limited to human, as we have also identified unique CGs in, among others, the mouse and drosophila genomes, indicating towards their functional relevance. In addition to protein evolution, CGs can be responsible for gene regulation by preventing the expression of at least one or more of the parent genes. The fact that around 18% of the CG transcripts are expected to undergo NMD clearly suggests that some of these transcripts exist only transiently, and that the predicted resultant proteins are never actually expressed.

Although more than 20% of the human protein-coding genes are capable of forming CGs, we could identify only 800 CGs in our analysis, which is the highest number of CGs shown so far in the entire human genome. The limitation to our approach for the identification of additional CGs is the availability of mRNA or EST sequences which support the CGs in the publicly available databases. The fact that most CGs, including some well-known examples such as *TNFSF12*-*TNFSF13* and *TMEM189*-*UBE2V1*, are supported by only a single mRNA or EST sequence indicates towards very low or tissue-specific expression of CGs. Therefore, more sophisticated techniques may be required to identify additional CGs in the human or other genomes.

To understand the complex systems biology of higher organisms it is critical to determine the role of CGs, such as has been done for other unusual types of transcripts, including ncRNAs identified in the human genome. However, the lack of a specific resource dedicated to these specialized CGs, compounded with the use of different terminologies for addressing them across currently available public genomic resources, greatly restricts this task. Therefore, the ConjoinG database should be particularly useful in this scenario. Identification of the underlying mechanism(s) controlling the formation of CGs in human and other vertebrate genomes remains, and is a target for future studies.

Clearly from this and other similar analyses, the same loci in a genome can operate in a multi-functional manner such that an exon of one gene can be the intron of another gene, or even an intergenic region for some other genes. These observations have once again put the definition of a ‘gene’ in question. Traditionally, a gene is a stretch of DNA that encodes for a type of protein that has a function in an organism. However, keeping in mind the other important regulatory functional roles performed by CGs, their annotation in the genome is equally important and should not be ignored.

## Methods

### Computational identification of conjoined genes

Alignments of ‘UCSC genes’, ‘Human mRNAs’ and ‘Spliced ESTs’ tracks (Human Genome assembly (hg18) March, 2006 build 36.1) were downloaded in GTF (Gene Transfer Format) format from the UCSC Genome Browser database for the human, mouse, dog, and fruit fly genomes. The reference sequences for these genomes were also downloaded from the same resource. An automated Perl algorithm “*Conjoin*” was developed for the identification of conjoined genes (see Text S1 for details). The algorithm is based on the positional comparison from the alignments of the known genes to the mRNA and EST sequences. The algorithm identifies all those mRNA and EST sequences which align to two or more different genes as defined in the NCBI RefSeq or UCSC Genes database. False positive cases arising out of misalignments and alternative splicing of the same loci were removed by manual curation.

### Experimental verification of conjoined genes

To validate the CGs, we used human poly(A)^+^ RNA from 16 different human tissues including brain, heart, kidney, liver, lung, pancreas, prostate, skeletal muscle, spleen, stomach, testis, uterus, fetal brain, fetal kidney, fetal liver, and fetal lung (Clontech Laboratories, Inc., Mountain View, CA, USA), which were reverse-transcribed with oligo(dT)_20_ using the ThermoScript RT-PCR System for First-Strand cDNA Synthesis (Invitrogen Corp., Carlsbad, CA, USA). Two sets of primers, external (first) and internal (nested) primers, were designed from the genomic sequences corresponding to the CG mRNAs and ESTs using the Primer3 [35] software. RT-PCR was basically performed with TaKaRa Ex Taq (Takara Bio Inc., Otsu, Shiga, Japan) and both first and nested primer sets to detect the expected CG mRNA or EST. The PCR products were introduced into the pT7Blue-2 T-Vector (Novagen, San Diego, CA, USA) and then transformed into *E. coli* strain, DH5 alpha. Colony PCR was performed using the *E. coli* clones carrying the PCR products originating from a few selected tissues in which CG sequences were expected to be transcribed. These PCR products were subsequently subjected to sequencing with the BigDye Terminator v3.1 Cycle Sequencing Kit (Applied Biosystems LLC, Foster City, CA, USA) on an Applied Biosystems 3130*xl* Genetic Analyzer and a 3730*xl* DNA Analyzer (Applied Biosystems LLC).

### Analysis of conjoined gene ORFs

For analysis of the splicing patterns and ORFs encoded by the CGs, only those candidates were selected for which at least one mRNA sequence was found so that a full-length sequence could be expected. Out of the 751 CGs, only 317 were found with at least one supporting mRNA sequence, for a total of 426 mRNA sequences. For the analysis of CG ORFs, the predicted proteins corresponding to all the parent genes and 264 out of 426 CG mRNAs were obtained from the NCBI GenBank database (http://www.ncbi.nlm.nih.gov/Genbank/). For the remaining CG mRNAs the longest ORF starting with a methionine, if found, was used. For 17 CG mRNAs, either reliable ORFs could not be predicted or the predicted proteins corresponding to the parent gene(s) were not found, so they were excluded from this analysis, leaving a total of 409 CG mRNAs (297 CGs). Alignments of the parent genes and CG proteins were generated using the Emboss Needle program (http://emboss.sourceforge.net/) with default options.

### Conservation of conjoined genes

To measure the conservation of CGs in other species, the mRNA and EST libraries of 23 other vertebrate genomes were downloaded from the UCSC Genome Browser database. BLAT (version 33) was used to align the ‘junction exons’ from the CGs to the vertebrate genome mRNA and EST sequences using an E-value cut off of less than 10^−6^. In addition, only matches where greater than 90% of the junction exon sequence was conserved with greater than 90% sequence identity were considered.

### Development of ConjoinG database

Open Source LAMP (Red Hat Enterprise Linux 4) Technology, Apache (version 2.2.8), MySQL (version 5.0.45), PHP (version 5.2.4), and Perl (version 5.8.5) were used for development of the GUI and back-end database called ‘Conjoindb’ (http://metasystems.riken.jp/conjoing/). The web server was developed using the Apache HTTP Server (version 2.2.8). Client-side scripting was done using XHTML, JavaScript, and AJAX, and server-side scripting was done using PHP and XML. The external application BLAST [36] was integrated for additional analysis.

## Supporting Information

Figure S1 Status of conjoined gene FPGT-TNNI3K in the NCBI Entrez Gene database, the UCSC Genome Browser, the Vertebrate Genome Annotation (Vega) database, the Ensembl Genome Browser, and the ConjoinG database. The CG FPGT-TNNI3K is not reported in either NCBI or UCSC, although both parent genes, FPGT and TNNI3K, are present (shown by red block arrows), while in Vega and Ensembl this CG is reported as TNNI3K (shown by red block arrow), and the parent gene FPGT is not present at all. The locus representing the FPGT gene in NCBI and UCSC is represented as a variant of TNNI3K in Vega and Ensembl.
(4.76 MB TIF)

Figure S2 Example of a false positive case due to gene name variants of the same gene on chromosome 2 (UGT1A complex locus). The members of this family have different names, so the mRNA or EST sequences aligning in this region are falsely predicted as possible CG candidates. For example, mRNA accession AF030310 (shown in red) will be predicted as a CG transcript combining many members of the UGT1A complex locus. Such false positive cases were removed during the manual curation step of our analysis.
(0.13 MB TIF)

Table S1 Alternative names used for conjoined genes.
(0.03 MB DOC)

Text S1 Identification of conjoined genes in the human genome.
(0.31 MB DOC)

Text S2 Functional analysis of the parent genes.
(0.25 MB DOC)

Text S3 Chromosomal distribution of the conjoined genes.
(0.10 MB DOC)

Text S4 Distribution of cis-regulatory elements in the upstream regions of CGs.
(0.06 MB DOC)
