# Supplementary figures and images for: Expression of Conjoined Genes: Another Mechanism for Gene Regulation in Eukaryotes
Source: PLoS One. 2010 Oct 12;5(10):e13284. doi: 10.1371/journal.pone.0013284 (PMC2953495; doi:10.1371/journal.pone.0013284)

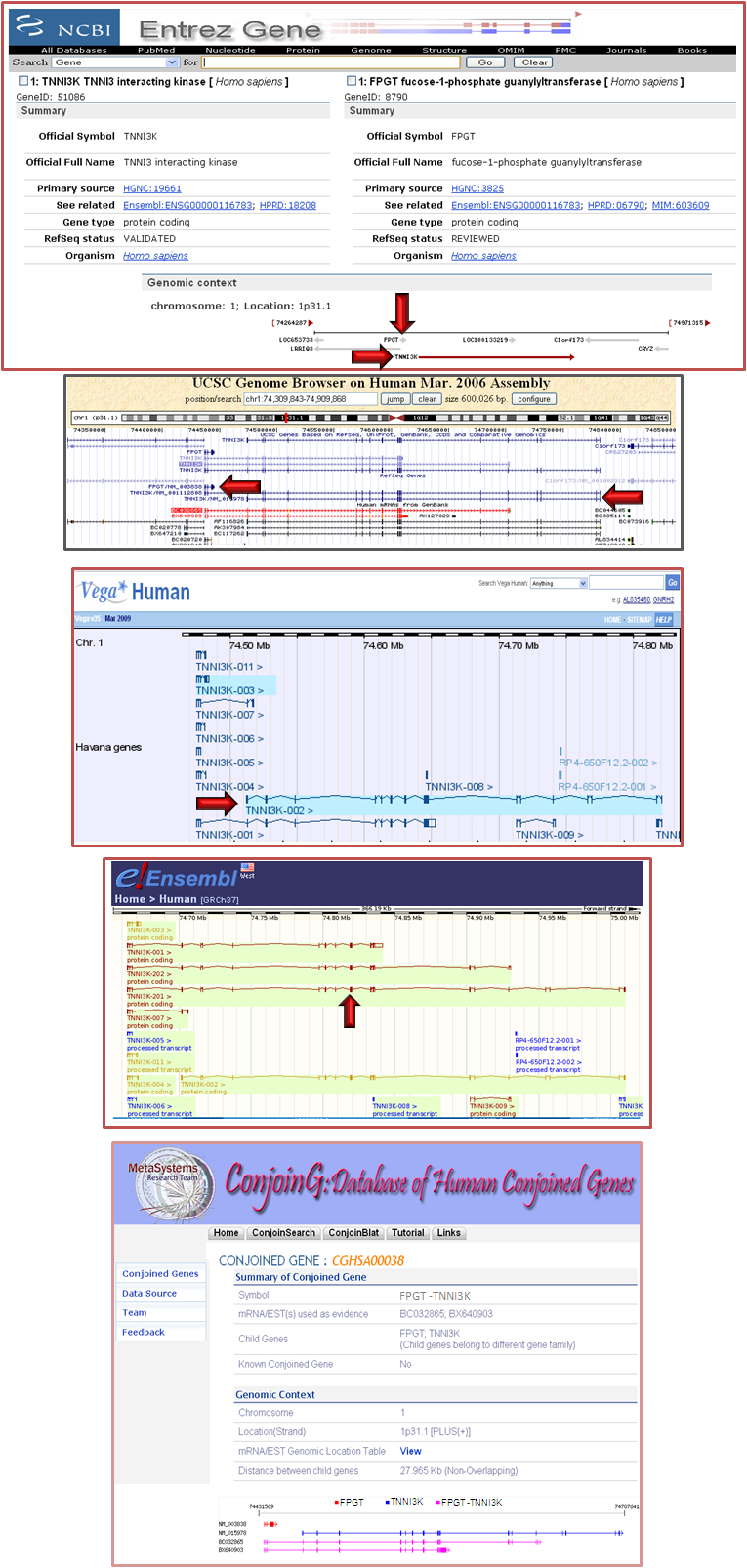

Supplement: Figure S1 — Status of conjoined gene FPGT-TNNI3K in the NCBI Entrez Gene database, the UCSC Genome Browser, the Vertebrate Genome Annotation (Vega) database, the Ensembl Genome Browser, and the ConjoinG database. The CG FPGT-TNNI3K is not reported in either NCBI or UCSC, although both parent genes, FPGT and TNNI3K, are present (shown by red block arrows), while in Vega and Ensembl this CG is reported as TNNI3K (shown by red block arrow), and the parent gene FPGT is not present at all. The locus representing the FPGT gene in NCBI and UCSC is represented as a variant of TNNI3K in Vega and Ensembl. (4.76 MB TIF) [file pone.0013284.s001.tif]

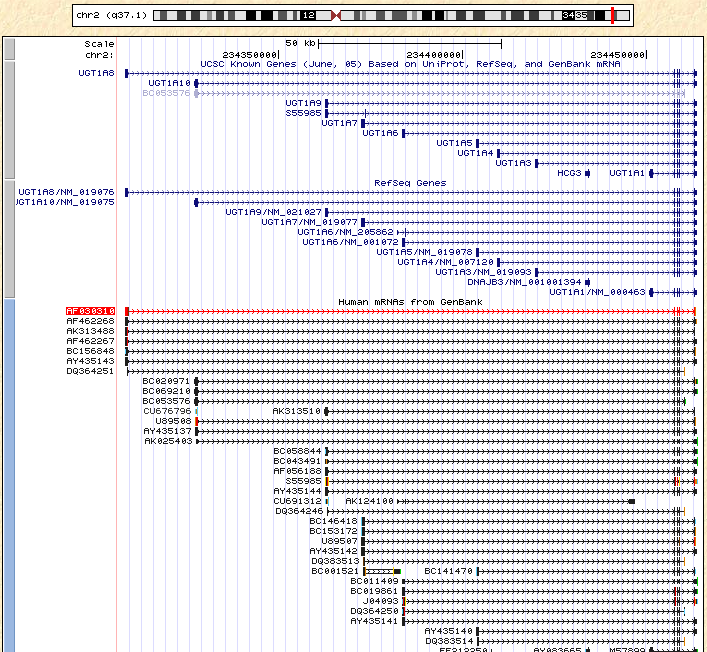

Supplement: Figure S2 — Example of a false positive case due to gene name variants of the same gene on chromosome 2 (UGT1A complex locus). The members of this family have different names, so the mRNA or EST sequences aligning in this region are falsely predicted as possible CG candidates. For example, mRNA accession AF030310 (shown in red) will be predicted as a CG transcript combining many members of the UGT1A complex locus. Such false positive cases were removed during the manual curation step of our analysis. (0.13 MB TIF) [file pone.0013284.s002.tif]
