# Supplementary material for: Expression of Conjoined Genes: Another Mechanism for Gene Regulation in Eukaryotes
Source: PLoS One. 2010 Oct 12;5(10):e13284. doi: 10.1371/journal.pone.0013284 (PMC2953495; doi:10.1371/journal.pone.0013284)
Supplement: Table S1 — Alternative names used for conjoined genes. (0.03 MB DOC) [file pone.0013284.s003.doc]

| **Alternative Name** | **Standard Definition** |  |
| --- | --- | --- |
| Fusion gene | Fusion genes are hybrid genes formed from two previously separate genes as the result of a translocation, interstitial deletion, or chromosomal inversion. |  |
| Chimeric proteins / Fusion proteins | Fusion proteins, AKA chimeric proteins, are proteins created through the joining of two or more genes which originally coded for separate proteins. However, not all conjoined genes form proteins, and are considered non-coding transcripts. |  |
| Read-through transcript | Read-through transcription is a well established process in higher eukaryotes, which commonly refers to genes having multiple polyadenylation sites leading to transcripts that extend for variable distances into the 3' flanking region of the gene. |  |
| Co-transcribed gene | Genes which are transcribed at the same time. The genes which co-transcribe may not necessarily form a single transcript containing parts of exons from each of the co-transcribing genes. |  |
| CoTIS | Co-Transcription and Intergenic Splicing refers to the phenomenon when two adjacent, independent genes may be co-transcribed and the intergenic region is spliced out so that the resulting fused transcript possesses exons from both genes. |  |
| Chimeric/chimaeric RNA | Chimeric RNAs refer to the following three kinds of transcripts: 1) transcripts encoded by two or more loci located within one chromosome but on different strands, 2) transcripts encoded by two loci located on different chromosomes, and 3) transcripts with shuffled exon order compared to genomic DNA sequences. |  |
|  | | |
| **Other Proposed Name** |  |  |
| Chimeric (fusion) transcript |  |  |
| Bridged gene |  |  |
| Spanning gene |  |  |
| Hybrid gene |  |  |
| Locus-spanning transcript |  |  |
| Fusion sequence |  |  |

**Supplementary Information Table S1: Alternative names used for conjoined genes.**
