# Supplementary material for: Expression of Conjoined Genes: Another Mechanism for Gene Regulation in Eukaryotes
Source: PLoS One. 2010 Oct 12;5(10):e13284. doi: 10.1371/journal.pone.0013284 (PMC2953495; doi:10.1371/journal.pone.0013284)

**Supplementary Information Text S1:**

***Identification of conjoined genes in the human genome***

To estimate the total number of CGs, an automated Perl algorithm “*Conjoin*” was developed and applied to the entire human genome (UCSC genome annotation database for the Mar. 2006 GenBank freeze assembled by NCBI (hg18, Build 36.1)). Figure A shows a flow chart detailing the steps of our algorithm. From the UCSC Genome Browser, alignments of the following tracks were downloaded and used as inputs for the *Conjoin* algorithm: UCSC known gene (track name: UCSC genes; table name: knowngenes), mRNA (track name: Human mRNAs; table name: all_mrna), and EST (track name: Spliced ESTs; table name: intronEST). The steps of the algorithm are as follows: (i) Retrieve the RefSeq accessions for all the “UCSC Known Gene IDs” using the file “knownToRefSeq.txt” from the UCSC sequence and annotation downloads server. Merge the Entrez Gene IDs for the RefSeq accessions of all the known genes using the UCSC file “knownToLocusLink.txt” (ii) For each genome the alignments of the known gene tracks are compared with those of the mRNA and EST tracks to identify the cases where two distinct known (parent) genes with different Entrez IDs form part of a single mRNA or EST accession. (iii) Based on the location of the reference coordinates of the parent genes with respect to each other, the selected mRNAs and ESTs are classified into ‘formed by non-overlapping’ or ‘formed by overlapping’ (including gene-within-gene and partially overlapping) parent genes. For mRNAs and ESTs formed by non-overlapping parent genes, those with at least one exon having a match of more than 30 bps from each parent gene are considered as possible CG candidates. A pre-processing step is included for mRNAs and ESTs formed by overlapping parent genes in which all overlapping exons are excluded from both parent genes. Those mRNAs and ESTs with at least one exon from the remaining unique exons having a match of more than 30 bps from each parent gene are considered as possible CG candidates.

For this analysis, running the “*Conjoin*” algorithm resulted in 623 and 942 CG candidates using human mRNA and EST databases, respectively. (iv) On merging the gene symbol information from the HUGO Gene Nomenclature Committee (<http://www.genenames.org/>), some transcripts were identified as formed from parent genes belonging to the same gene family. The products of genes from the same gene families usually show more than 40% amino acid sequence identity. Thus, the mRNA or EST sequences spanning two or more such parent genes have a higher possibility of misalignment or alignment at more than one location. In addition to these, due to the general poor quality of EST sequences and their relatively small size, a similar situation may arise when aligning ESTs to the whole genome. Also, alternatively spliced variants of same gene loci are sometimes assigned similar gene names, but carry different Entrez Gene IDs. A few CG candidates resulted from such parent genes. To remove the false positives arising from duplicated regions in the genomes, genes belonging to the same gene family, or variants of the same gene loci, a final step of manual curation was performed. This resulted in 751 conjoined genes supported by a total of 1,466 mRNA and EST transcripts connecting 1,451 distinct parent genes in the entire human genome. Interestingly, 88 out of 751 CGs (12%) were supported by more than one mRNA or EST transcript, while the majority (663, 88%) were represented by only one supporting transcript. Based on the HGNC gene symbols of the parent genes, the CGs were classified as belonging to either the same, different, or unknown gene families. 83 out of 751 CGs (11%) were formed by parent genes belonging to the same gene family.

**Figure A: Flow chart detailing the steps of the *Conjoin* algorithm for the identification of conjoined genes in the human genome.** The March 2006 human genome assembly (hg18, UCSC Genome Browser database) was used for this analysis.


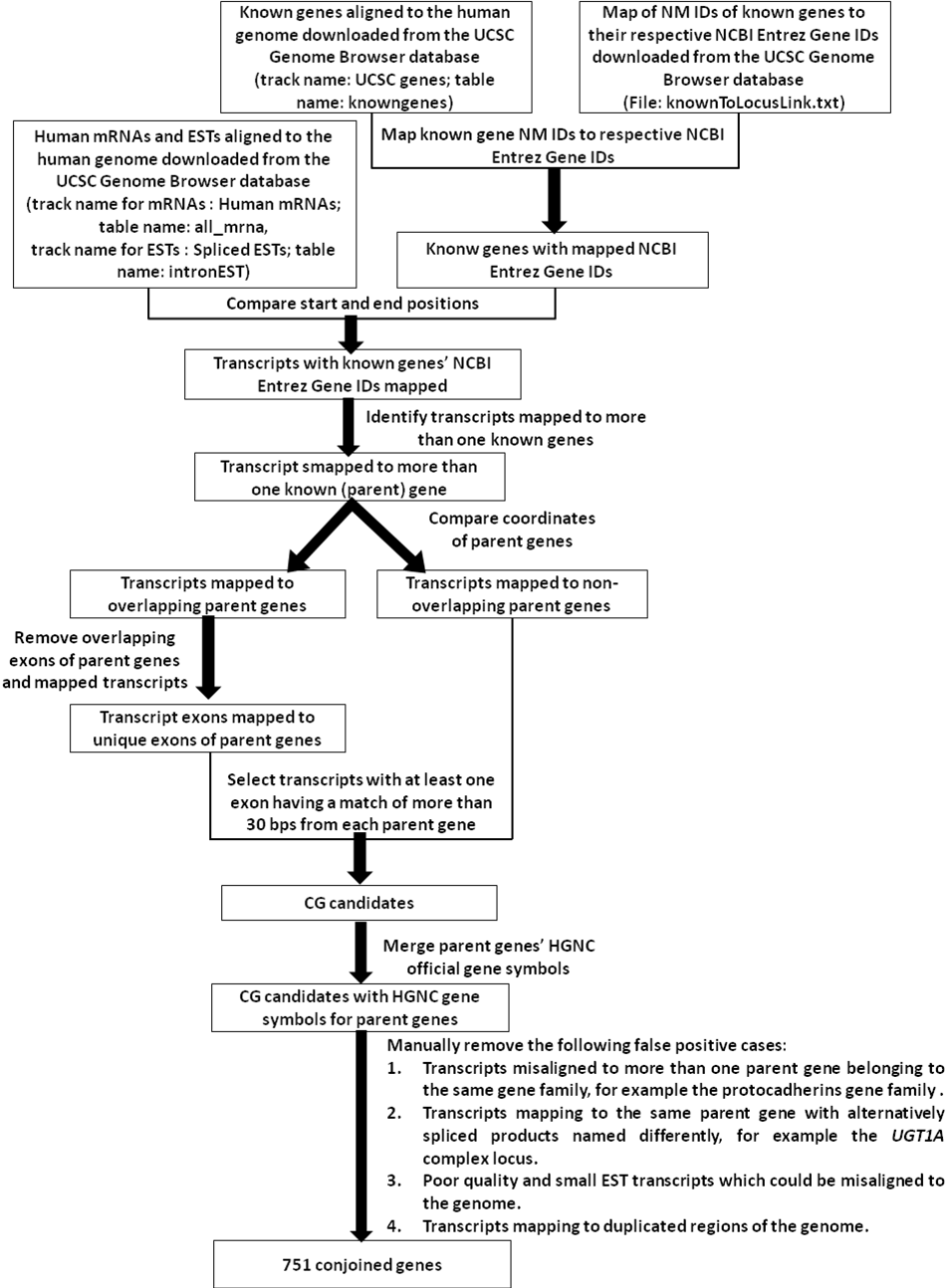

Supplement: Text S1 — Identification of conjoined genes in the human genome. (0.31 MB DOC) [file pone.0013284.s004.doc]
