# Supplementary material for: Expression of Conjoined Genes: Another Mechanism for Gene Regulation in Eukaryotes
Source: PLoS One. 2010 Oct 12;5(10):e13284. doi: 10.1371/journal.pone.0013284 (PMC2953495; doi:10.1371/journal.pone.0013284)

**Supplementary Information Text S2:**

***Functional analysis of the parent genes***

To explore if any functional preference exists among the parent genes, they were classified into various functional categories under the four major functional classes as defined in the clusters of orthologous groups for eukaryotes (KOGs) (1). This task was performed using BLAST with an E-value cut-off of less than 10^-6^ against all the protein sequences classified under KOGs and subsequent extraction of the KOGs classification for the best match. Functional information could be obtained for 66% (954) of the parent genes. Of these, 31% belonged to the “Poorly Characterized” functional class. The remaining parent genes were distributed among the “Cellular Processes and Signaling” (36%), “Information Storage and Processing” (16%), and “Metabolism” (16%) functional classes (figure A). Because the most abundant class of genes in the human genome is “Cellular Processes and Signaling” (figure B), the formation of CGs by parent genes belonging to this functional class by chance cannot be ruled out. Yet, it is noteworthy that this functional class contains those genes which have more recently evolved and are more specific to the higher eukaryotes (2). Thus, formation of CGs is a potential mechanism for the regulation of genes involved in the communication process, a function almost exclusive to higher organisms such as human.

**References:**

1. Tatusov, R. L., Fedorova, N. D., Jackson, J. D., Jacobs, A. R., Kiryutin, B., Koonin, E. V., Krylov, D. M., Mazumder, R., Mekhedov, S. L., Nikolskaya, A. N. *et al.* (2003) The COG database: an updated version includes eukaryotes. *BMC. Bioinformatics.* 4:41. Epub;%2003 Sep 11.: 41.

2. Andrade, M. A., Ouzounis, C., Sander, C., Tamames, J., & Valencia, A. (1999) Functional classes in the three domains of life. *J. Mol. Evol.* 49: 551-557.

**Figure: A)** **Distribution of parent genes into KOG functional categories. B) Distribution of all the human genes into KOG functional categories.** *Blue represents Cellular Processes and Signaling genes* [D - Cell cycle control, cell division, chromosome partitioning; M - Cell wall/membrane/envelope biogenesis; N - Cell motility; O - Posttranslational modification, protein turnover, chaperones; T - Signal transduction mechanisms; U - Intracellular trafficking, secretion, and vesicular transport; V - Defense mechanisms; W - Extracellular structures; Y - Nuclear structure; Z - Cytoskeleton], *cyan represents Information Storage and Processing genes* [A - RNA processing and modification; B - Chromatin structure and dynamics; J - Translation, ribosomal structure and biogenesis; K - Transcription; L - Replication, recombination and repair], *pink represents Metabolism genes* [C - Energy production and conversion; E - Amino acid transport and metabolism; F - Nucleotide transport and metabolism; G - Carbohydrate transport and metabolism; H - Coenzyme transport and metabolism; I - Lipid transport and metabolism; P - Inorganic ion transport and metabolism; Q - Secondary metabolites biosynthesis, transport and catabolism], *and grey represents Poorly characterized genes* [R - General function prediction only; S - Function unknown].

**A).**


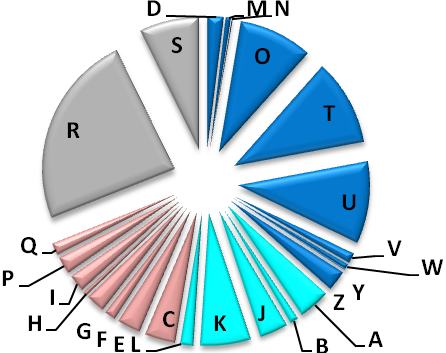


**B).**


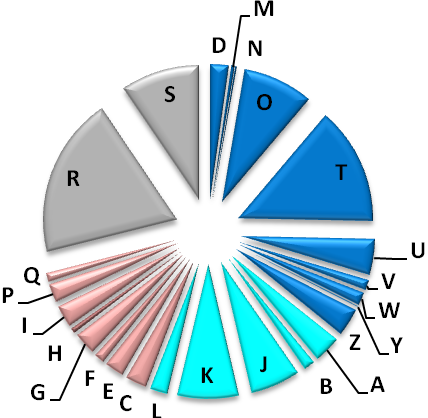

Supplement: Text S2 — Functional analysis of the parent genes. (0.25 MB DOC) [file pone.0013284.s005.doc]
