# Supplementary material for: Expression of Conjoined Genes: Another Mechanism for Gene Regulation in Eukaryotes
Source: PLoS One. 2010 Oct 12;5(10):e13284. doi: 10.1371/journal.pone.0013284 (PMC2953495; doi:10.1371/journal.pone.0013284)
Supplement: Text S3 — Chromosomal distribution of the conjoined genes. (0.10 MB DOC) [file pone.0013284.s006.doc]

**Supplementary Information Text S3:**

***Chromosomal distribution of the conjoined genes***

Conjoined genes are formed by pairing of parent genes on the same chromosomal strand, usually within 10 kb apart from one another. Thus, it would be interesting to determine if any correlation exists between the total number of genes and the number of CGs on each chromosome. The non-parametric Spearman Rank Correlation test was used to determine the degree of correlation (association) between the two variables. As shown in the figure below, larger chromosomes with more genes carry a higher number of CGs as compared to those with fewer genes (Spearman Rank Correlation Coefficient (R) = 0.8813, Number of data points (N) = 24, p-value <= 1 (Standard score (Z) = 4.2266)). Because mRNA and EST sequences were used in our analysis as evidence for CGs, the number of CGs identified may also be affected by the amount of transcript information available. A large number (88%) of CGs were found to be represented by only one connecting mRNA or EST sequence. This indicates toward either weak expression of these genes or limited availability of transcript information for the genome. Although a vast amount of transcriptome information is available for the human genome, not all transcripts have yet been identified. Thus, it is of interest to see if any correlation exists between the number of CGs identified and the actual number of transcripts available for each chromosome. The Figure below shows that chromosomes with more transcript information also carry more CGs (R = 0.8909, N = 24, p <= 1 (Z = 4.2725)). Another feature affecting the number of CGs on the chromosomes could be the average distance between the genes. In some chromosomal regions the genes are evenly spaced, whereas in others they are clustered together. There are also some regions, such as gene deserts, on the chromosomes which are completely void of any annotated gene. Interestingly, as the average distance between genes decreases the number of CGs increases (Figure below) (R = -0.5372, N = 24, p <= 1 (Z = -2.5762)). And, most CGs are formed by genes which are less than 10 kb apart. Since CGs are formed by connecting two or more parent genes occurring on the same strand, the frequency of genes occurring on the same strand of a chromosome could also affect the number of CGs. This frequency showed a positive trend with the number of CGs, but no significant correlation could be observed.


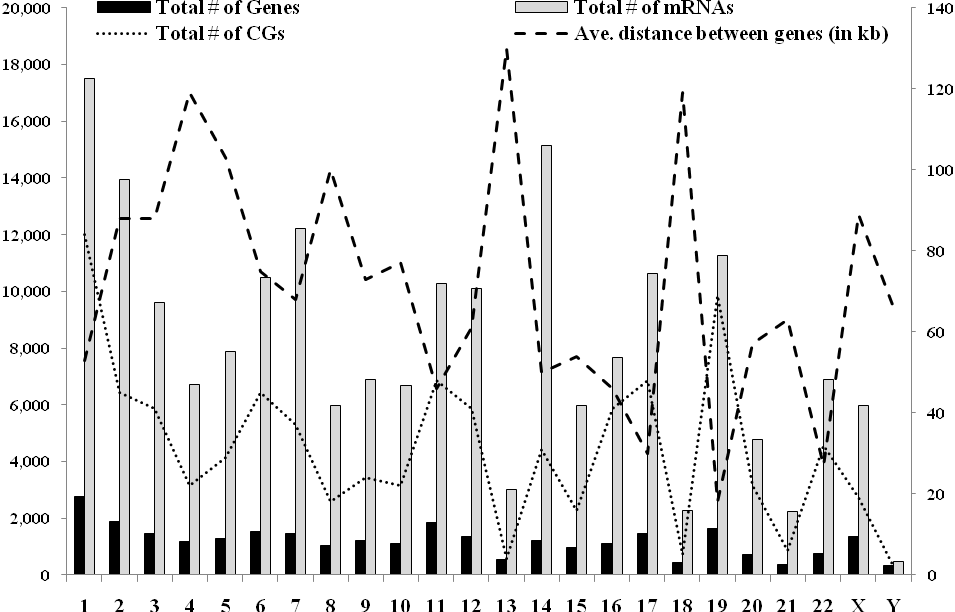


**Figure: Chromosomal distribution of conjoined genes.** The number of CGs increases significantly with the number of genes on a chromosome (Spearman Rank Correlation Coefficient (R) = 0.8813, Number of data points (N) = 24, p-value <= 1 (Standard score (Z) = 4.2266)). It also exhibits a positive correlation with the total number of mRNA transcripts mapped to a chromosome (R = 0.8909, N = 24, p <= 1 (Z = 4.2725)). However, the number of CGs shows an inverse relationship with the average distance between the genes on the chromosomes (R = -0.5372, N = 24, p <= 1 (Z = -2.5762)).
