# Supplementary material for: Expression of Conjoined Genes: Another Mechanism for Gene Regulation in Eukaryotes
Source: PLoS One. 2010 Oct 12;5(10):e13284. doi: 10.1371/journal.pone.0013284 (PMC2953495; doi:10.1371/journal.pone.0013284)
Supplement: Text S4 — Distribution of cis-regulatory elements in the upstream regions of CGs. (0.06 MB DOC) [file pone.0013284.s007.doc]

**Supplementary Information Text S4:**

***Distribution of cis-regulatory elements in the upstream regions of CGs***

Detailed analysis for a few important cis-regulatory elements such as simple and complex repeats, and CpG islands which are known to play important roles in gene regulation, was carried out in the 2 kb upstream region of the conjoined genes and the results were normalized with respect to a control dataset. GC composition was also measured. For the control dataset, pairs of adjacent genes were selected which lie in opposite orientation on the same chromosome and which are at least 10 kb apart (since most CGs were found to be formed by parent genes which are less than 10 kb apart). No distinct patterns of occurrence of these elements could be observed in the upstream regions of the conjoined genes with respect to the control dataset (Figure S4), except for the simple repeats (Table S4). The control dataset was found to harbor more simple repeats than in the upstream regions of the conjoined genes. This indicates that the regulation of CGs may not be carried out by cis-regulatory elements; rather, some other trans-acting factors may be governing their expression.

**Figure S4:** Distribution of cis-regulatory elements in the 2 kb upstream regions.

**In the 2 kb upstream regions of the conjoined genes**

**Between the upstream (5’-) and downstream (3’-) ends of the conjoined genes, including the intronic regions**

**CGs**

**Control dataset**

**CpG Islands Repeat contentGC %**

**Table S4:** Distribution of simple repeats in the 2 kb upstream regions.

|  | **Total number of sequences** | **Number of Simple repeats** | **% of Simple Repeats** |
| --- | --- | --- | --- |
| **Conjoined genes** | 561 | 80 | 14.3 |
| **Control dataset** | 1,423 | 595 | 41.8 |
